# Health Economics of Dengue: A Systematic Literature Review and Expert Panel's Assessment

**DOI:** 10.4269/ajtmh.2011.10-0521

**Published:** 2011-03-04

**Authors:** Mark E. Beatty, Philippe Beutels, Martin I. Meltzer, Donald S. Shepard, Joachim Hombach, Raymond Hutubessy, Damien Dessis, Laurent Coudeville, Benoit Dervaux, Ole Wichmann, Harold S. Margolis, Joel N. Kuritsky

**Affiliations:** Pediatric Dengue Vaccine Initiative, International Vaccine Institute, Seoul, Republic of Korea; Centre for Health Economics Research and Modeling Infectious Diseases, Centre for the Evaluation of Vaccination, Vaccine and Infectious Disease Institute, University of Antwerp, Antwerp, Belgium; Centers for Disease Control and Prevention, Atlanta, Georgia; Heller School, Brandeis University, Waltham, Massachusetts; Initiative for Vaccine Research, World Health Organization, Geneva, Switzerland; GlaxoSmithKline Biologicals, Wavre, Belgium; Sanofi Pasteur, Lyon, France; Université Catholique de Lille, Lille, France

## Abstract

Dengue vaccines are currently in development and policymakers need appropriate economic studies to determine their potential financial and public health impact. We searched five databases (PubMed, EMBASE, LILAC, EconLit, and WHOLIS) to identify health economics studies of dengue. Forty-three manuscripts were identified that provided primary data: 32 report economic burden of dengue and nine are comparative economic analyses assessing various interventions. The remaining two were a willingness-to-pay study and a policymaker survey. An expert panel reviewed the existing dengue economic literature and recommended future research to fill information gaps. Although dengue is an important vector-borne disease, the economic literature is relatively sparse and results have often been conflicting because of use of inconsistent assumptions. Health economic research specific to dengue is urgently needed to ensure informed decision making on the various options for controlling and preventing this disease.

## Introduction

Dengue fever is a rapidly increasing public health problem in tropical and subtropical regions with a large percentage of the world's population at risk.^1^ Resource-poor countries are particularly hard hit because of inadequate public health infrastructure, lack of resources to combat the vector, and limited health care services to manage cases.^2^ The most recent estimates suggested 50 million infections and 20,000 deaths occur each year.^1^ Several tetravalent dengue vaccine candidates are in phase 1 and 2 clinical trials and one candidate has entered a large-scale efficacy and safety trial.^3^,^4^ Depending on the results of these and future clinical trials, a dengue vaccine could be licensed in the next 5 years.

The Pediatric Dengue Vaccine Initiative (PDVI) is a product development partnership^5^ whose goal it is to accelerate development, evaluation, and introduction of dengue vaccines in endemic countries.^6^ To understand the economic impact of the disease and to strategically plan for further research, PDVI conducted a systematic review of the literature and convened a panel of experts to assess the results and provide recommendations on the priorities and methodology for conducting further research, especially disease burden and cost-of-illness studies, comparative analyses, and modeling for planning vaccine introduction strategies.

## Materials and Methods

### Expert panel.

In April 2008, the PDVI sponsored a meeting of experts (coauthors) in health economics or dengue in Antwerp, Belgium, to review the existing literature on dengue health economics, identify future research needs, and provide recommendations on priorities and methodology for conducting further research. Future research was prioritized by the panel on the basis of their expert opinion and past experience after considering estimated study costs, surmised interest to decision makers on dengue vaccine development and introduction, and the assumption that an approved dengue vaccine would be available within 5 years. Need-based priority was rated numerically, 1 = urgent, 2 = needed, 3 = optional. The panel then assigned a time period during which the needed studies should be started so the results are available for planning when a dengue vaccine becomes available; 1–2 years, 2–4 years, and 5–6 years. Because the interest in economic studies varies based on the needs of different stakeholders in dengue vaccine, interest in each type of study result was rated from high to low on the basis of the expert opinion of the panel.

### Literature search.

After designing an agreed search strategy, inclusion/exclusion criteria, and quality rating, in March 2008 (updated in January 2010), MEB conducted searches of published literature for economic studies of dengue in the following data bases, without restriction to publication year or language: U.S. National Library of Medicine and the National Institutes of Health Medical (PubMed) (1966–2009); Excerpta Medica Database (EMBASE) (1983–2009); Latin American and Caribbean Health Sciences Database (LILAC) (1967–2009); American Economic Association's electronic bibliography of economic literature (EconLit) (1969–2009); and World Health Organization (WHO) library (WHOLIS) (1985–2009). The search criteria combined the medical subject headings (MeSH) “dengue,” “economics,” “health economics,” “costs and cost analysis,” “cost of illness,” “quality of life,” with the text words “economic,” “cost,” “best practice analysis,” “budget impact,” “DALY,” and “QALY.” Unpublished reports were also included if they were identified in a database (i.e., LILAC, WHOLIS, EconLit) or referenced in a publication identified in the initial search. Abstracts and full text of identified manuscripts were reviewed and the following inclusion criteria were applied: 1) analyzed both costs and clinical outcomes, 2) provided detailed methods, and 3) involved original data analysis. Excluded were reviews, editorials, and studies involving previously published data. The quality of data was assessed (by MEB) according to the scale developed by Sackett and others^7^ and recommended by the York Centre^8^ but modified for dengue (Table 1). After piloting, the data abstraction instrument was applied to all included studies (MEB). The variables abstracted became the column headings for Tables 2 and 3. The full text of included studies was circulated to all coauthors before convening the expert panel and after the update search was completed. Results and discussion of the studies were synthesized by MEB and circulated for additional comments to all coauthors.

### Classification scheme.

We categorized identified publications according to economic methods (macroeconomic versus microeconomic) and study objectives (e.g., quantify disease burden or assess the impact interventions [comparative analysis]).

Disease burden studies were categorized by the metric used to quantify burden—non-monetized units (e.g., DALY^9^) or monetized units (e.g., dollars); the latter being classified as a cost-of-illness (COI) study. The COI studies were categorized by level or perspective of the payer (e.g., government, healthcare system, household) and government perspective was further subdivided into public health costs and budget impact of vaccine introduction.

Comparative analyses were categorized by type of intervention (i.e., vaccine versus vector control) and the value used to make the comparisons: 1) cost-effectiveness analyses used unvalued or natural health gains (e.g., cases of dengue, deaths averted, life-years gained); 2) cost-utility analyses valued outcomes in units that reflected measures of morbidity and mortality, such as quality (QALYs) or disability-adjusted life years (DALYs); and 3) cost-benefit analyses converted health outcomes into monetary units to enable comparisons between interventions in the health and other sectors (e.g., education) to estimate return on investment.

## Results

**Figure 1.**
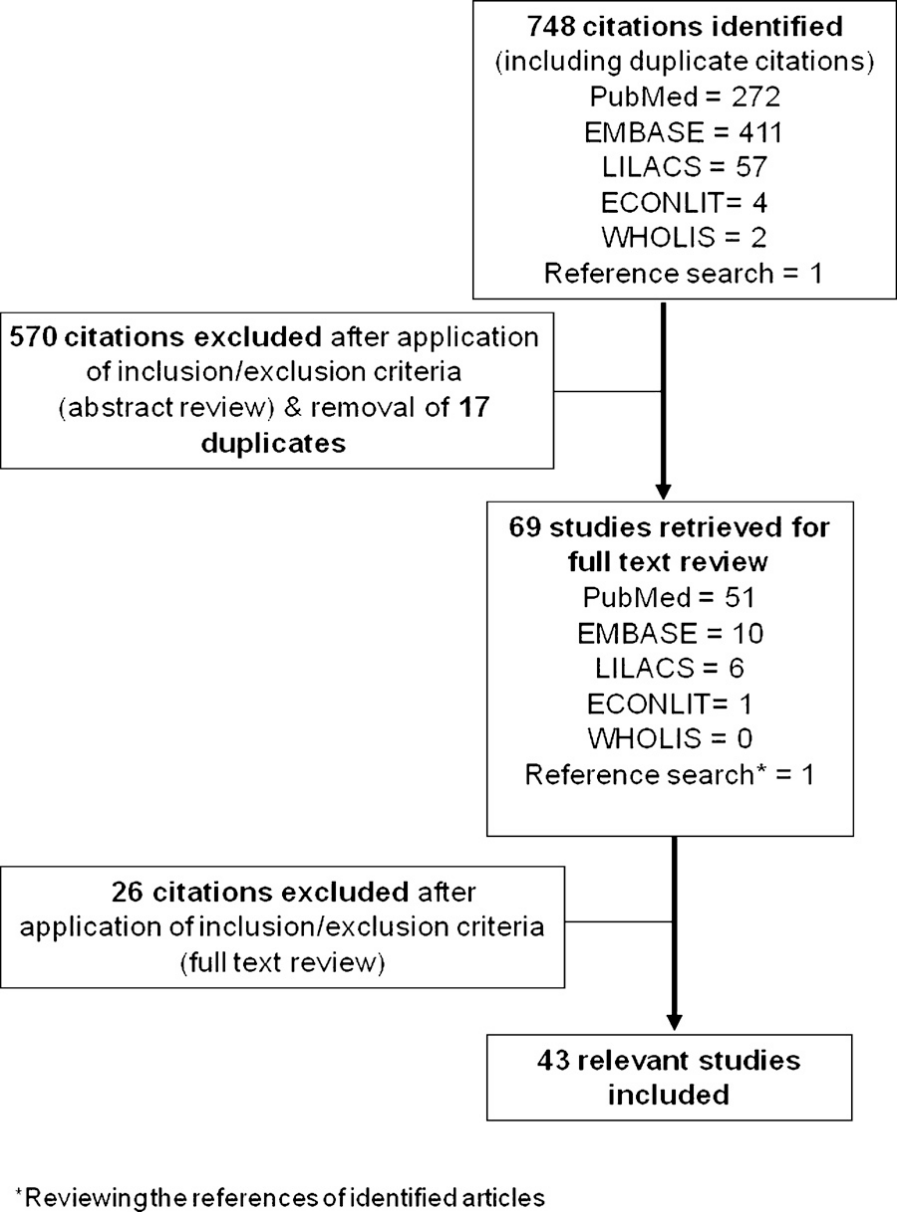
Results of systematic literature search and evaluation of identified studies.

Our search indentified 748 citations (Figure 1). Of these, 43 were dengue-related economic studies that fulfilled the inclusion criteria.^10^–^55^ Forty-one used microeconomic methods: 32 report economic burden of dengue (Table 2),^10^–^12^,^15^–^18^,^20^–^32^,^34^–^37^,^41^,^42^,^46^,^47^,^49^–^55^ and nine are comparative economic analyses assessing various interventions (Table 3).^13^,^14^,^33^,^38^,^39^,^43^–^45^,^48^ The remaining two were a willingness-to-pay (WTP) study and a policymaker survey.^19^,^40^

### Disease burden.

#### Without monetization.

Eight studies expressed the dengue burden in DALYs^10^,^17^,^22^,^29^,^31^,^32^,^34^,^53^–^55^ This measure has been used since the early 1990s to determine disease burden and facilitate disease comparison and prioritization.^9^,^29^,^53^,^54^ The DALY is a summary measure essentially combining the occurrence and duration of a disease, with its lethality and severity (expressed in a “disability score,” a higher score signifying worse health). However, there has been great variability in reported dengue disease burden using DALYs, which has occurred for several reasons. Early on only the more severe form of dengue, dengue hemorrhagic fever (DHF), was measured or reported but not the less severe dengue fever (DF).^56^ As a result, only DHF incidence was used in early DALY calculations.^53^ Subsequently, cohort studies in Asia showed that DHF represented a small proportion of symptomatic infections,^57^–^59^ and its sole use to define a case would underestimate dengue cases by a factor of 2- to 10-fold.

A second factor related to variability in reported dengue disease burden has been inconsistent application of disability scores, which are a measure of disease severity and essential to the DALY calculation. Early scores were probably too low, ranging from 0.172 to 0.211 (at that time equivalent to uncomplicated malaria or a radius fracture in a hard cast), whereas the duration of illness of 30 days^29^,^53^,^54^ was too long.^10^,^57^–^59^ An early study that used only nationally reported DHF cases used a disability score of 0.22 but shortened the duration of illness to 20 days,^17^ which reduced the DALY estimate by 30%.

In 2004, WHO revised the burden estimates for dengue.^55^ For DHF, the disability score was increased to 0.5 but the duration of illness was shortened to 11 days, which resulted in the same DALYs obtained in 1990 when the disability score was ∼0.2 and duration of illness was 30 days. More importantly, DF was included in the new estimates, with an assigned disability score of 0.211 and illness duration of 5.5 days. In comparison, an uncomplicated febrile episode of malaria, which is often in the differential diagnosis of DF because of the similarity in clinical presentation and severity, was increased from its 1990 disability score of 0.21 to a score of 0.471, more than doubling the DALY estimate.^60^

Because patients with DHF require hospitalization and cannot care for themselves, Meltzer and others used a 0.81 disability score^34^ (equivalent to a severe migraine^53^ or diseases that interfere with one's ability to care for oneself) but used shorter durations of each type of illness^34^ to be consistent with clinical data,^47^,^61^ which more than doubled the estimated DALYs (Table 2). Subsequent studies have also used this higher disability score for DHF.^10^,^22^,^32^ Meltzer and others also documented that non-hospitalized cases, which typically are limited to DF, account for the greater portion of dengue disease burden,^34^ a finding confirmed by subsequent studies.^10^,^12^,^20^,^32^ However, even in countries or regions where non-hospitalized DF is reported, under-reporting is still significant.^62^

Luz and others reported that from 1986 through 2006, deaths accounted for the majority of the remaining disease burden caused by dengue after DF in Brazil, and that this proportion was increasing.^32^ Luz and others also reported that dengue disease burden was greatest at the city level followed by state and national levels.^32^ Although dengue is primarily a disease of urban centers,^2^ its disease burden in rural areas has been increasing.^63^–^65^

The duration of disability during dengue was not fully examined in the identified studies. A prospective study by Anderson and others determined the duration of measurable fever in hospitalized and non-hospitalized patients rather than the subjective history of fever.^10^ The average duration of fever was 6.35 and 5.32 days, respectively, for the two groups. Lum and others examined how dengue affected quality of life as measured by impact on daily activities or health domains (e.g., self care, mobility, cognition) using the WHO World Health Survey^66^ and visual analog scale (VAS) from the EuroQol EQ-5D.^67^ They found quality of life was impaired for 9 days among non-hospitalized patients and 13 days for hospitalized patients. At its lowest value, the VAS score was reduced to about 40% of that of its highest value, with slightly lower values for hospitalized versus ambulatory patients, and for adults versus children. Of eight health domains evaluated, an average of 5.0 were affected among non-hospitalized patients, and 6.2 among hospitalized patients.^31^ An advantage of this approach versus DALYs is that the patients were surveyed regarding how they experienced their disease, rather than that experts rated the severity of the disease stage. It was not clear, however, who served as respondent for children (i.e., a parent, a health care provider, or the study investigator).^68^ Moreover, although this was the first study to conduct a daily assessment of dengue on the quality of life, DALYS were not calculated—limiting comparability with previous studies.

Although the goal of using DALYs is to compare and prioritize diseases based on a common metric, in the case of dengue significant revision in the scoring has occurred since the original effort was undertaken, because disease burden studies aided by improved diagnostics raised awareness of the true breadth and spectrum of symptomatic dengue. Still, even the most recent estimates by WHO, underestimate dengue disease burden by using disability scores half those that are used by author researchers in the published literature. New disease burden estimates from WHO are expected soon and may be more consistent with contemporary estimates.

#### With monetization.

##### Government perspective.

Thirteen studies reported the cost of illness of dengue from the government perspective (Table 2)^12^,^16^,^20^,^23^,^30^,^35^–^37^,^41^,^42^,^46^,^49^,^51^; eight also included costs from both the healthcare system and household perspective. The majority was conducted in Latin America and over 10 years ago.^20^,^23^,^36^,^41^,^42^,^46^,^49^,^51^ Okanurak and others showed a significant difference in dengue cost of illness between children (0–15 years of age) and adults (> 15 years of age),^37^ which is consistent with clinical studies that showed children are less likely than adults to be symptomatic but more likely to have severe disease.^69^,^70^ Okanurak and others also documented a significant difference in cost of illness between a large referral center and provincial or community hospitals, although only three facilities were included in the study.^37^ Adam and others suggested that multiple facilities of the same type should be studied to define the degree and magnitude of cost variability.^71^

All studies itemized cost, but there was variation in what costs were included. Anderson and others found that transportation to healthcare facilities was a significant contributor to outpatient costs, but this was not included in all studies.^20^,^23^,^37^,^49^,^51^ Most studies included days of lost wages,^23^,^49^ and some included the value of days children were absent from school.^12^,^23^,^46^,^49^ The most inclusive COI studies were reported by Armien and others and Suaya and others. The Armien and others study^12^ was part of a larger eight country study coordinated by Suaya and others.^46^ Although the same COI is reported for Panama in both studies, Armien and others also estimated the cost of surveillance, laboratory testing, and vector control.^12^,^46^ Both studies also included lodging, food, and the cost of transportation to medical facilities for both patients and any accompanying or visiting family member, lost wages and productivity, which was assigned to used and unemployed persons and all family members caring for the patient.^12^,^46^ Additionally, the use of multiplication factors to account for under-reporting of cases to national surveillance systems had a major impact on aggregated cost estimates, whereas the inclusion of private healthcare providers was associated with substantially higher costs per case.^12^,^26^,^46^,^50^

In dengue endemic countries, transmission occurs year round with a seasonal peak incidence (dengue season). In addition, cyclical epidemics occur at 3–9 year intervals, but high rates of transmission usually continue during the inter-epidemic periods.^72^ All studies except that by Canyon,^16^ report data from epidemic years, and none provided data from non-epidemic years in the same country. Dengue epidemics could result in great variation in government and healthcare expenditures, including actual costs per case. For example, during a typical non-epidemic year in Brazil, 200,000 dengue cases were reported but during the 2002 epidemic this number rose to 800,000.^73^ In addition, Siqueira and others^73^ showed the proportion of dengue cases that required hospitalization increased during epidemics.^73^ While this could be caused by increased disease severity from biologic factors (virus or host),^72^ it may be behavioral. Several household studies showed increased parental fear and concern when a child developed a fever during a dengue epidemic.^28^,^47^ In addition, the expert panel suggested that rationing of scarce resources during an epidemic may actually reduce the cost per patient.

#### Public health costs.

Five of the 13 studies reported government costs of dengue prevention and control.^30^,^35^,^36^,^41^,^42^ Lok itemized the costs of vector control in Singapore, including person hours, equipment, and pesticides.^30^ The Brazilian Ministry of Health^31^ included costs of education, laboratory surveillance, and legislative activities.^35^ Two Pan American Health Organization (PAHO) reports provided the annual cost of vector control for 23 countries in their region.^41^,^42^ A study by Nathan evaluated the *Aedes aegypti* control program in 24 Caribbean and selected neighboring countries; all in the PAHO region.^36^

Lok identified a common problem in allocation of costs for vector control programs; rarely were specific staff or equipment solely designated for dengue.^30^ As a result, if dengue were controlled through other means (e.g., vaccine), the associated reallocation of resources to other vector control activities would lead to savings in opportunity costs, but not necessarily in financial expenditures. This is particularly true when other diseases (e.g., yellow fever, Chikungunya) are transmitted by the same vector (e.g., *Aedes* mosquitoes) necessitating continued *Aedes* control efforts. This is observed for yellow fever and Japanese encephalitis, both vaccine preventable diseases that include vector control programs as an integral part of their prevention and control plans.^74^,^75^ However, a true saving in vector control activities might occur if vaccination eliminated dengue epidemics with their attendant intensified activities and expenditures.^76^,^77^

#### Budget impact.

No reports were identified detailing country-specific cost estimates for dengue vaccination programs as has been done for other vaccine preventable diseases.^78^,^79^ Such preplanning has been shown to facilitate introduction of a new vaccine.^80^

#### Healthcare system perspective.

Four studies reported dengue COI from the perspective of the healthcare system. Harling and others assessed the impact of travel associated disease cared for in the United Kingdom healthcare system, where dengue accounted for 2% of the total cost.^24^ The study was limited because costs for hospitalized patients were estimated on a per-day basis without itemization. Wong and others used diagnostic billing codes (i.e., Australian National Diagnosis-Related Group (AN DRG) version 3.1 [1996]) to estimate the direct medical costs for hospitalized dengue patients.^52^ Dengue was also the main focus of studies by Añez and others (Venezuela)^11^ and Garg and others (India)^21^ who used an average cost of hospitalization after summing accumulated costs associated with the study facilities. Añez and others multiplied this estimate by the reported number of cases,^11^ whereas Garg and others used a multiplication factor to account for under-reporting.^21^ The latter study had several weaknesses including, the use of a multiplication factor and age distributions derived from Thailand instead of India.^21^ Because the age distribution and standards of care for dengue treatment often differ by region,^63^,^81^,^82^ their estimates may not be valid. Furthermore, Garg and others used an average duration of illness based on all febrile illnesses rather than dengue, which have been shown to have a significantly longer duration of fever.^10^

#### Household perspective.

Dengue household impact studies have documented not only the financial burden of dengue but also intangibles such as emotional stress for an entire household.^25^,^27^,^28^,^47^,^50^ Dengue is among the infectious diseases that can cause unexpected catastrophic medical costs for families living in low-income countries (a catastrophic cost has been defined as ≥ 40% of the capacity to pay, on the basis of a household's non-subsistence effective income.^83^ These costs are even greater when multiple cases occur in the same household, which is common during epidemics.^25^,^47^,^50^ Parents often express fear for their children with regard to dengue, which likely affects health-seeking behavior and increases their willingness to spend and incur debt for perceived higher quality healthcare services.^50^ The issue of debt is important because this has been shown to persist for more than a year in households where children have been hospitalized for dengue.^26^,^32^,^50^,^84^ Loss of assets and ongoing debt are rarely accounted for in COI studies. Such variations have also been described for other diseases, such as malaria.^85^,^86^ Challenges with these studies include the uneven and seasonal changes in income that can alter impact^85^,^86^ and complexities of estimating the value of bartered goods and services.

### Comparative analyses.

#### Vaccines.

There were two published studies of potential cost-effectiveness of dengue vaccination compared with either vector control or case management (Table 3).^43^,^44^ In 1993 Shepard and Halstead estimated the cost-effectiveness of immunization compared with vector control and case management in the context of two levels of healthcare system development—“developed” or “undeveloped” (e.g., Thailand and Laos, respectively).^43^ Base-case assumptions included a 3.8% dengue incidence, 1.2 billion people at risk of infection, a two-dose vaccine regimen at US$17.50/dose with a US$0.50 administration cost per dose.^43^ Costs of alternative control methods were US$0.46 per capita for chemical vector control and US$2.25 for environmental vector control. Direct medical costs for hospitalized DHF cases were estimated at US$200; non-hospitalized cases were not included. The authors found a dengue vaccine would be cost-effective (average US$1,440 per DALY saved and US$92,461/death averted) in countries with poorly developed healthcare delivery systems, but case management would be more cost-effective in countries classified as “developed.”^43^ A sensitivity analysis indicated vaccine would become cost-effective in developed countries at ≤ US$7.00/dose.

A subsequent study (including the same authors) in 2004 analyzed 10 Southeast Asian countries with an estimated population of 529 million and dengue incidence based on WHO reporting.^44^ Other assumptions included a two-dose regimen costing US$0.50/dose in the public sector and $10/dose in the private sector, US$3.50/dose for vaccine administration, 1% annual disease incidence, treatment costs (direct and indirect) of US$139 for DHF and US$4.29 for DF, and vector control costs of US$0.02–$3.56 per capita.^44^ This study found vaccine to be potentially cost-effective (average US$50 per DALY, 52% because of reduction in premature mortality), at US$7.64/dose (weighted average for public and private sector including administration costs).^44^ The difference in results of the two studies reflects wide differences of input assumptions, which makes it difficult to compare the results of the studies. The WHO has recently proposed guides that may help standardizing such analyses more.^87^,^88^

#### Vector control.

There have been seven additional comparative studies that focused on vector control for dengue prevention (Table 3).^13^,^14^,^33^,^38^,^39^,^45^,^48^ The study by Arthur D. Little, Incorporated reported that eradication of *Aedes aegypti* was more cost-effective than on-going control in Latin America.^13^ Although the studies by McConnell and others, Suaya and others, and Orellano and Pedroni also estimated the potential economic impact of control programs on disease incidence,^33^,^38^,^45^ the studies by Osaka and others, Baly and others, and Tun-Lin and others prospectively compared outcome measures in intervention and control communities interventions.^14^,^39^,^45^ Osaka and others used disease incidence as the outcome measure,^39^ whereas Baly and others and Tun-Lin and others used larval indices.^14^,^48^ Because of its randomized and multicenter, multicountry design, Tun-Lin and others was the most powerful study and effectively showed that targeted larval control was at least as effective as non-targeted control but at a lower cost except where the intervention incorporated social mobilization.^48^ However, while the use of larval indices are correlated with the prevalence of human dengue infections,^89^ outbreaks still occur at what are considered low larval indices.^90^

### Stated preferences research.

In the one identified WTP study, Palanca-Tan asked 205 persons living in metropolitan Manila to consider a single dose, safe and efficacious dengue vaccine.^40^ Their willingness to pay for such a vaccine was elicited by a dichotomous choice approach,^91^ with an average that ranged from US$27 to $32.^40^ The WTP studies also captured psychological and social aspects of health outcomes that cannot be assessed through traditional cost-of-illness studies (e.g., satisfaction the individual derives from using the resources or the value attached to future use).^88^ Perhaps, more important than preferred cost, this type of study can estimate public demand for a vaccine.^19^

Dengue can become such an important political issue that government officials may lose favor as a result of choices made during dengue epidemics.^92^–^95^ Indeed, policymakers in four Southeast Asian countries (Cambodia, Indonesia, Philippines, and Vietnam) expressed a high level of concern regarding DF and a great need for a vaccine.^19^ In addition, they indicated that disease surveillance studies, in-country vaccine trials or pilot projects, and studies on the economic burden of dengue and the cost-effectiveness of dengue vaccines were necessary for informed decision making regarding vaccine introduction.^19^ Surveys of policymakers may be informative in predicting public support for vaccine introduction and their importance for decision making should not be underestimated.^19^ For example, public concern about disease awareness rather than considerations of cost-effectiveness drove recommendations for use of meningococcal vaccine in college students in the United States and other countries.^96^,^97^

## Discussion

On the basis of the review of available studies, the expert panel recommended types of economic studies they thought would fill information gaps related to development and introduction of dengue vaccines (Table 4). In addition, they assigned priorities and suggested a time frame during which these studies should be completed; primarily influenced by estimated time for first approval of a dengue vaccine. Finally, the panel indicated their perception of importance of these studies to decision-makers involved in development or introduction of a dengue vaccine. Below are specific comments for the recommended studies (Table 4).

### Disease burden.

Accurate disease incidence data are required to provide robust estimates of disease burden across the regions where dengue is endemic. National dengue surveillance programs are designed to detect disease trends and detect outbreaks and their sensitivity and specificity is often affected by program budgets. For this reason, further studies are required to quantify under detection and under-reporting. Dengue incidence studies should be prospective, performed in defined populations that are representative of the community, and include a wide range of ages. Febrile illness should be the starting point to ascertain dengue cases, which should be defined by well-established and validated laboratory methods that include molecular diagnostics for DENV and/or DENV NS1 antigen detection and immunoglobulin M (IgM) anti-DENV.^98^

The results from these studies can then be compared with national surveillance data gathered in the same region to provide reasonable multiplication factors to account for under-reporting. Alternatively, a more rigorous method for estimating the degree of disease under-reporting is to perform a capture–recapture study.^62^

Additional prospective COI studies are needed that include representative sampling from each type of heath care facility in both the public and private sectors, preferably over multiple years. Because DHF and DF are not classified in a uniform way across all dengue endemic regions,^99^ the expert panel recommended that cases be categorized by outcomes, such as non-hospitalized, hospitalized, or death. Adjustment is needed for age-specific differences in rates of symptomatic disease in children and adults. Many studies have used age categories of 0–15 years and > 15 years, which was considered the minimum acceptable age stratification by the expert panel.

The panel stressed that future studies should clearly indicate assumptions, include costs, and use dengue- and country-specific data. Itemized lists of costs appropriate for micro-costing of programs have been published,^100^ and WHO has developed guides for economic studies.^87^,^88^ Comprehensive multiperspective studies that document the total cost of illness but also allow for analysis from each perspective are useful to decision makers. The exceptions are budget impact studies of vaccination programs conducted from the government perspective, which are used to determine best approaches to vaccine implementation.

### Comparative analyses.

Additional comparative analyses to estimate the potential economic impact of a dengue vaccine are a high priority. The experts recommended cost-effectiveness or cost-utility analyses to avoid the inherent difficulties of cost-benefit analyses associated with converting benefits, including lives saved, into monetary units. These studies should include: 1) clearly defined and referenced assumptions; 2) country-specific or internationally vetted costs of vaccination and potential vaccine related adverse events, and added costs of post-implementation surveillance to monitor safety and efficacy of a new vaccine; 3) estimated impact on mortality, as policymakers may expect this to be high, despite the fact that dengue is a relatively low mortality disease; 4) reporting of cost-effectiveness in natural units such as hospitalizations averted, deaths averted, life-years gained, and as DALYs averted or QALYs gained; and 5) a discount rate of 3% for both costs and effects as recommended in WHO guides.^88^ The meeting participants agreed that recently developed WHO Guides for standardization of economic evaluations of immunization programs and economic consequences of disease should be followed to allow comparability between studies.^87^,^88^ Furthermore, a number of specific issues related to model-based economic evaluation of vaccines as opposed to curative drugs, are relevant but outlined elsewhere.^53^,^101^

Designing cost-effectiveness studies before a vaccine has been fully evaluated requires assumptions about variables such as efficacy and effectiveness, dosage and costs. The panel advised the following: 1) assume first generation tetravalent dengue vaccines would require at least a two-dose regimen, and likely three doses; 2) determine the threshold price for a vaccine to be cost-effective rather than assigning a specific cost, because it is difficult to predict cost (public and private) for a vaccine that has not been marketed; and 3) conduct sensitivity analyses on epidemiological, effectiveness, and cost estimates to determine the uncertainty of the cost-effectiveness estimates.

A static cost-effectiveness model developed for pandemic influenza,^102^ has been shown to stimulate interest and further country-specific economic research (Meltzer MI, personal communication) and could have the same effect for dengue. Dynamic transmission models are also needed and should account for serotype-specific immunity, herd protection, vector-host interactions, seasonal variations in disease transmission, and age-specific differences in disease incidence and severity. These models in turn should be coupled with economic models to assist in choosing the most efficient and cost-effective options for intervention.

### Stated preferences research.

The panel recommended that future studies of this type include an assessment of acceptance of a potential vaccine with varying levels of effectiveness and price, and questions that allow comparisons with prevention of other diseases.

### Limitations.

By its very nature, the systematic literature review in this work captured mainly published studies, and is therefore subject to publication bias. We aimed to minimize potential exclusions of valuable sources by searching the reference lists of retained studies (Figure 1) to identify relevant books, unpublished data, evaluations, and dissertations. Furthermore, we did not restrict the review by language. That said, only English, Spanish, French, and Portuguese were encountered during the search.

## Conclusion

Although dengue is an important vector-borne disease, the economic literature is relatively sparse and results have often been conflicting because of use of inconsistent assumptions. This review of the literature captures the available information at a point in time. We presume that as new information becomes available it will be added to this information base. Health economic research specific to dengue is urgently needed to ensure informed decision making on the various options for controlling and preventing this disease—an option, which in the not too distant future, is likely to include vaccination.

## Figures and Tables

**Table 1:** Quality grading scale for economic studies of dengue^7^,^8^

Quality score	Description
I	Evaluation of important alternative interventions comparing all clinically relevant outcomes (e.g., non-hospitalized, hospitalized, or dengue hemorrhagic fever [DHF], and death) against appropriate cost measurement, and including a clinically sensible sensitivity analysis
II	Evaluation of important alternative interventions comparing a limited number of outcomes against appropriate cost measurement, but including a clinically sensible sensitivity analysis
III	Evaluation of important alternative interventions comparing all clinically relevant outcomes against inappropriate cost measurement, but including a clinically sensible sensitivity analysis
IV	Evaluation without a clinically sensible sensitivity analysis
V	Expert opinion with no explicit critical appraisal, based on economic theory

**Table 2:** Summary of dengue disease burden studies

Study first author; quality score; reference*	Study period; location; total cases: estimated (actual)	Level; included markets†	Cost per year (cost per case)	Costs included or characteristics of disability caused	Comment on comparability	Strengths
*Without monetization*						
World Health Organization; II^29^,^53^,^54^	1990; world; 415,000	N/A‡	142 DALYs§/M¶ 2000: 62 DALYs/M 2010: 28 DALYs/M 2020: 14–38 DALYs/M	Age-specific disability scores: 0–4 years = 0.211, –14 years = 0.195, > 15 years = 0.172; duration: DHF‖ = 30 days; years of life lost to death = 37	(1) Only included DHF and deaths. (2) Data based on epidemiologic assessment in 1990 and projected to 2020. (3) Projections assumed diseases like dengue would continue a downward trend in incidence.	(1) First use of DALYs. (2) Used same disability score as malaria. (3) Is a compendium of disability estimates for 200 conditions using the same methods and allowing comparisons between diseases and available for comparison.
World Health Organization; I^55^	2004; world; 9 million	N/A	104 DALYs/M	Disability score: DF**7 = 0.2, DHF = 0.5; duration: DF = 5.5 days, DHF = 30 days; years of life lost to death = 33	Source of incidence date: DengueNet††	(1) Same authors as 1990 study. (2) Reassessment of epidemiologic and other assumptions from 1990 study.
Cho-Min-Naing; II^17^	1970–1997; Myanmar (range: 391–13,085)	N/A	90–97 DALYs/M	Disability score = 0.22; duration: DHF = 20 days; years life lost to death = 37	Included only DHF and deaths	First country-specific study.
Meltzer; I^34^	1984–1994; Puerto Rico; total estimated but not reported (total varied annually; range: 1,865–18,880)	N/A	658 DALYs/M	Disability score = 0.81; duration: DF = 4 days, DF with hemorrhage = 10 days, hospitalized DF or DHF = 14 days; years of life lost to death = 44	(1) Duration of illness was based on novel case classification (e.g., non-hospitalized; non-hospitalized with hemorrhage; hospitalized). (2) Age specific multiplication factor used for first time: (10 × reported cases for ages 0–15 years; 27 × for ages > 15 years.	(1) Classification system includes non-hospitalized cases and attempts to address variability in disease severity. (2) Considered children (0–15 years) and adults (> 15 years) separately.
Gubler; I^22^	1955–1996; Southeast Asia, Latin America, India, China, Caribbean; estimates used‡‡	N/A	575 DALYs/M	Disability score = 0.81; duration: DF = 6 days, DHF = 14 days; years of life lost to death = 28	(1) Relied on reported cases that rarely included non-hospitalized cases. (2) Cases from India and China excluded. (3) Total cases were a moving average and with exact values not stated. (4) Multiplication factors: 102×–105× reported cases.	(1) Used two independent methods to estimate total cases. (2) Considered children (0–15 years) and adults (> 15 years) separately.
Anderson; I^10^	1998–2002; Kamphaeng Phet Province, Thailand (cohort study: *N* = 2,114)	HH; Pub & Priv	465 DALYs/M (US$10§§/ DF case, US$1032/hosp. DF case, US$1039/ hosp. DHF case)	Direct; indirect; disability score = 0.81; duration: DF = 4.36 days, hospitalized DF = 6.35 days, hospitalized DHF = 8.41 days	(1) Limited to children (5–15 years). (2) Duration of illness defined as observed febrile period rather than the subjective report of recovery, which is the standard method. (3) Cost of transport to and from medical facilities not included.	(1) Prospective cohort study design. (2) Included non-hospitalized and hospitalized cases. (3) Compared with non-dengue febrile illnesses
Lum; II^31^	2005; Klang Valley, Malaysia (survey: *N* = 207)	N/A	Determined effect on health domains¶¶ (EuroQuol).‖‖ Of 8 health domains measured: non-hospitalized = 5.0 affected; hospitalized cases = 6.2 affected	Duration: non-hospitalized = 9 days, hospitalized = 13 days	(1) Did not use age cutoff of 15 years used in all previous publications. (2) New classification of severity: DF, DF with plasma leakage, and DHF. (3) No comparisons of metric used (EuroQuol to DALYs)	(1) Reports primary data on cost. (2) Considered children (0–12 years) and adults (> 12 years) separately.
Luz; I^32^	1986–2006; Brazil (range: 1,570–794,219)	N/A	Rio de Janeiro (city) 226 DALYs/M, Rio de Janeiro (state) 197 DALYs/M, Brazil (country) 87 DALYs/M	Disability score = 0.81; duration: DF = 2–7 days, DHF = 10–18 days; Years of life lost to death = 32–44	(1) Dengue incidence steadily increased over the study period, taking the mean DALY estimates over the study period results in underestimate of the current burden. (2) Disease incidence and reporting may vary widely resulting across a large country.	(1) Compared DALY calculated for Rio de Janeiro (city), Rio de Janeiro (state), for Brazil (country). (2) Included non-hospitalized and hospitalized cases. (3) Included all ages
*With monetization: government perspective*						
Von Allmen; IV^51^	July–December, 1977; Puerto Rico (11,840)	Gov, HS & HH; Pub & Priv	US$1·2 million (US$23–36/case)	Direct; indirect; surveillance; prevention	(1) Patient costs based on reference costs for consultation and hospitalization (dengue vs. any fever not specified). (2) Direct medical cost did not include travel to appointment. (3) Indirect cost included only ill worker. (4) Data collected during year of an outbreak.	Cost of lost days of school reported but not included in the reported total.
Guzmán; IV^23^	1981; Cuba; 344,203	Gov, HS & HH; Pub	US$103 million (US$299/case)	Direct; indirect; prevention	(1) Patient costs based on reference costs for consultation and hospitalization (dengue vs. any fever not specified). (2) Direct medical cost did not include travel to appointment. (3) Prevention costs included only vector control activities and not surveillance. (4) Special costs associated with 148 reported fatalities were not included. (5) Data collected during year of an outbreak.	(1) First data from Cuba. (2) Considered children (0–15 years) and adults (> 15 years) separately.
Ferrando; IV^20^	August–December, 1994; Nicaragua; 60,916 (14,442)	Gov, HS & HH; Pub & Priv	US$2.7 million (US$44/case)	Direct; indirect; surveillance; prevention	(1) Patient costs based on reference costs for consultation and hospitalization (dengue vs. fever of any cause). (2) Direct cost did not include travel to appointment. (3) Indirect cost included did not include cost of care taker. (4) Data collected during year of an outbreak.	(1) Cost of lost days of school reported. (2) Considered children (0–15 years) and adults (> 15 years) separately. (3) Includes non-hospitalized disease.
Okanurak; IV^37^	1994; Thailand; 51,688	Gov, HS & HH; Pub	US$12.6 million (Bangkok: US$118/child US$161/adult, Suphan Buri: US$102/child, US$138/adult)	Direct; indirect; surveillance; prevention; years of life lost to death not reported	(1) Only hospitalized DHF patients included. (2) Data collected during year of an outbreak.	(1) Reports primary data collected on cost. (2) Data gathered from regional, provincial, and community facilities. (3) Considered children (0–15 years) and adults (> 15 years) separately.
Valdés; IV^49^	January–May, 1997; Santiago, Cuba; 5,245 (3,012)	Gov, HS & HH; Pub	US$0.3 million (US$594/case)	Direct; indirect; prevention; surveillance	(1) Included only hospitalized cases. (2) 12 deaths occurred but not included in the cost. (3) Indirect included only days of lost work for adults. (4) Data collected during year of an outbreak.	Multiplication factor based on serosurvey data.
Armien; I^12^	2005; Panama; 32,900 (5,489)	Gov, HS & HH; Pub & Priv	US$16.9 million (US$332/non-hospitalized case; US$1,065/ hospitalized case)	Direct; indirect; prevention; surveillance	(1) Direct costs included food, lodging, and “miscellaneous expenses” for any family member visiting the patient. (2) Indirect costs included not only primary care takers time but every family reporting taking part in the care of sick family member. (3) Replaced reported wages of under used with minimum wage of country. (4) Hospitalized cost based on six cases. (5) Data collected during year of an outbreak.	Reports primary data collected on cost data.
Canyon; V^16^	1879–2005; Australia; total not given	Gov, HS & HH	US$2.7 million	Direct; indirect; prevention	(1) Methods are not fully detailed. (2) The data are not reported by year.	Provides information trends in transmission of the last century.
Suaya; I^46^	Brazil, Cambodia, El Salvador, Guatemala, Malaysia, Panama, Thailand, Venezuela,	Gov, HS & HH	US$851 million for all 8 countries (mean: US$248/non-hospitalized case; US$571/hospitalized case)	Direct; indirect	(1) Cost data for Panama was previously published by Armien and others.^24^ (2) Protocols varied from country to country. (3) Direct costs included food, lodging, and “miscellaneous expenses” for any family member visiting the patient. (4) Indirect costs included not only primary care takers time but every family reporting taking part in the care of sick family member. (5) Replaced reported wages of under used with minimum wage of country. (6) Data collected during year of an outbreak.	(1) Provides updated cost data from 6 countries and new data from El Salvador, Guatemala, and Malaysia. (2) Costs estimated were averaged across the sites and reported individually.
*With monetization: government perspective, public health costs*						
Lok; IV^30^	1981; Singapore; total not given	Gov	US$2.2 million (US$1.39/capita)***	Vector control	Included only the cost of vector control program.	Itemized budget that included equipment maintenance, utility, and, transport broken down by health district and including central government, and specific to *Aedes aegypti*.
Nathan; IV^36^	1990; Caribbean††† total cases not given	Gov	US$11.3 million	Vector control	Program evaluation.	(1) No data on human disease provided. (2) Each country's budget listed separately.
Pan American Health Organization; IV^41^	1995; PAHO countries;‡‡‡ total not reported	Gov	US$104 million	Vector control; surveillance; prevention	Complete cost itemization not done.	(1) No data on human disease provided. (2) Each country's budget listed separately.
Pan American Health Organization; IV^42^	1996, 1997; PAHO countries;‡‡‡ total not reported	Gov	1996: US$330 million 1997: US$679 million	Vector control; surveillance; prevention	Complete cost itemization not done.	(1) No data on human disease provided. (2) Each country's budget listed separately.
MOH Brazil; IV^35^	2002; Brazil; total cases not reported	Gov	US$362 M***	Vector control; surveillance, prevention	(1) Only in Portuguese. (2) Reported costs in Riel.	Itemized by state.
*WITH MONETIZATION :HEALTHCARE SYSTEM PERSPECTIVE*						
Añez; IV^11^	1997–2003; Zulia, Venezuela; 2,187–8,295	HS, HH	US$193,000 (US$43/non-hospitalized case, US$173/hospitalized case)	Direct; indirect	(1) Used an average cost of consultation in 2004 for direct medical cases (did not include transport to healthcare facility). (2) Average cost of hospital day estimated to be the cost of all treatments and ordered tests for an average hospitalized fever patient and multiplied by 7 days, the average duration of hospitalization. (3) Did not include costs of professional staff services; utilities; etc	Considered children (0–15 years) and adults (> 15 years) separately.
Harling; IV^24^	1998–1999; England and Wales (*N* = 421 hospitalized cases)	HS	US$26,000/year (US$640/hospitalized case)***	Direct	(1) Study to assess the economic impact of travel associated infection. (2) Cost based on estimated cost per day for hospitalization without itemization. (3) Dengue was not the primary focus of the study: only 2% (8/421) admissions were dengue. (4) Limited to hospitalized cases.	Reports primary data collected on cost.
Wong; IV^52^	2004–2005; Singapore (*N* = 46,249 hospitalized cases)	HS; Pub	(US$252–341/hosp. case)***	Direct	(1) Study completed to evaluate the impact requiring publication of billing charges for specific diagnoses. (2) Direct medical costs were not itemized. (3) Limited to patients age ≤ 60 years. (4) Dengue was not the primary focus of the study. (5) Limited to hospitalized cases.	(1) Analysis included all five public hospitals in Singapore. (2) Allows comparison of hospitalize cost of dengue to 28 common diagnosis-related groups.
Garg; I^21^	September–November 2006; India; 123,170–332,559 (12, 317)	HS & HH; Pub & Priv	US$27.4 M (US$432/hospitalized case)	Direct; indirect; years of life lost to death =10–30	(1) Costs extrapolated from a single tertiary private hospital. (2) Cost for consultation and hospitalization not specific for dengue. (3) Data collected during year of an outbreak.	(1) Considered children (0–15 years) and adults (> 15 years) separately. (2) Assumptions regarding under-reporting may not be valid.
*WITH MONETIZATION :HOUSEHOLD PERSPECTIVE*						
Torres; IV^47^	1990; Lares, Puerto Rico (Survey: *N* = 97)	HH	(US$125/case)	Indirect	(1) Cost assigned only to days of lost work and other forms of productivity. (2) Data collected during year of an outbreak	(1) Average cost based on retrospective data collected from 97 households. (2) Included investigation of psychological impact of dengue (e.g., stress). (3) Assigned value to losses incurred by unsalaried workers (e.g., housewives, service exchanges, etc.)
Clark; I^18^	2001; Kamphaeng Phet Province,Thailand; 1,244,090 (124,409)	HH	(US$61/case) 427 DALYs/M	Direct; indirect; disability score = 0.81 duration: non-hospitalized = 4 days; hospitalized case = 9.1 days; years of life lost to death = 44	(1) Limited to children (0–15 years). (2) Limited to hospitalized patients.	(1) Average household impact based on interview and review of hospital records from 204 retrospectively identified cases. (2) Accounted for the impacted of multiple cases within in the same house.
Van Damme; IV^50^	2001; Banteay Meanchey Province, Cambodia (Survey: *N* = 72)	HH; Pub & Priv	(US$0–460/case)	Direct	Limited to children (0–15 years).	(1) Average cost based on retrospective data collected from 72 households. (2) Included investigation of incurred debt. (3) Documented the differences in health care costs between public and private providers.
Jacobs; IV^27^	2001–2002; Takeo Province, Cambodia (Survey: *N* = 404)	HH; Pub	(US$21/hospitalized case)	Direct; indirect	(1) Dengue was not the primary focus of the study: Study completed to evaluate the economic impact of user fees for hospitalization on households. (2) Indirect cost included only loss of missed wages.	(1) Reports primary data collected on cost. (2) Documents negative impact of user fees and explores the lasting debt that follows hospitalization.
Khun; IV^28^	March 2003–February 2004; Kampong Cham Province Cambodia (Survey: *N* = 19)	HH; Pub & Priv	(US$7.5/ hospitalized case)	Direct	(1) Cost data not systematically presented. (2) Study focused on health seeking behavior and indebtedness resulting from dengue.	(1) Reports primary data. (2) Qualitative methods to collect data included key informant interviews, focus group discussions, in-depth interviews and open-ended questionnaires, and ongoing observations.
Harving; IV^25^	2005; Ho Chi Minh City, Vietnam (Survey: *N* = 175)	HH; Pub & Priv	(US$6/case)	Direct; indirect	(1) Limited to children (0–15 years). (2) Limited to hospitalized DHF patients.	Reports primary data collected on cost.
Huy; IV^26^	2006; Kampong Cham Province Cambodia (Survey: *N* = 30)	HH; Pub & Priv	(US$15.4/ non hospitalized case, US$40.1/ hospitalized case)	Direct; indirect	Limited to children (0–15 years).	(1) Included an assessment of frequency and amount of debt incurred per illness. (2) Reimbursement included in the estimates. (3) Documented the differences in health care costs between public and private providers. (4) Results compared with an equal number of non dengue fever cases. (5) Cases were laboratory confirmed.

* See Table 1.

† Level or perspective of economic study (Gov = government; HS = health sector; HH = household); Markets on which cost data was collected: Pub = public; Priv = Private.

‡ N/A = not applicable.

§ DALYs = disability adjusted life-years.

¶ M = million population.

‖ DHF = dengue hemorrhagic fever.

** DF = dengue fever.

†† WHO dengue surveillance database available at http://www.who.int/csr/disease/dengue/denguenet/en.

‡‡ Estimates were used and reported for each year for the 40-year study period and each region. The data was too extensive to list here; the reader is referred to the original reference.

§§ US$ = Unites States dollars.

¶¶ Health domains are specific activities routinely carried out on a daily basis (e.g., self care, mobility, cognition).

‖‖ EuroQuol is a standardized visual analog scale on patients can quantify the level of reduction in ability to accomplish activities in various health domains.

*** Converted to US$ using historical exchange rate.

††† Anguilla, Antigua/Barbuda; Aruba, Bahamas; Barbados; Belize; Bermuda; Bonaire; British Virgin Islands, Cayman Islands, Dominica, Dominican Republic, Grenada, Jamaica, Martinique, Montserrat, St. Kitts, St. Lucia, St. Maarten, St Vincent, Suriname, Trinidad and Tobago, Turks and Caicos.

‡‡‡ Argentina, Bolivia, Brazil, Paraguay, Aruba, Colombia, Ecuador, Peru, Venezuela, Costa Rica, El Salvador, Guatemala, Honduras, Mexico, Nicaragua, Anguilla, Antigua and Barbuda, Barbados, Cuba, Dominica, Grenada, Monserrat, St. Lucia, St Vincent and the Grenadines, Trinidad and Tobago.

**Table 3:** Summary of dengue related comparative economic analyses

Study first author; quality score; reference*	Study type;† study period; location	Interventions compared	Costs included	Assumptions	Results
*Vaccines*					
Shepard; II^43^	CEA; not specified (publication date,1993); Central & South America, Caribbean, Southeast Asia	Vaccine; clinical management; vertical vector control (pesticide); environmental vector control (breeding site reduction)	Direct; vaccine administration; vector control; disease surveillance; vaccine development;	(1) Outcomes evaluated: reduction in DHF,‡ death. (2) Duration = 9 days. (3) Incidence = 0.0078/year. (4) Case-fatality rate = 0.058. (5), Treatment cost = US$200.§ (6) Vector control costs: Chemical = US$0.46/capita, Environmental = US$2.25. 7), Vaccine: Two-dose regimen; 95% effective; 73% vaccine coverage; US$17.50/dose + US$0·50/admin + US$2.40/healthcare contact.	In a country in which the health system is not developed (e.g., Laos), a dengue vaccination program would be cost-effective. In a country in which the health system is developed (e.g., Thailand), case management is most cost-effective approach. If cost for a dengue vaccination series dropped to US$7or case management rose to US$438/case, then vaccination would be more cost effective.
Shepard; I^44^	CEA; not specified (publication date, 2004); Southeast Asia	Vaccine; clinical management;	Direct; indirect	(1) Outcomes evaluated: reduction in DF, DHF, and death. (2) Duration: DF = 5.5 days, DHF = 9 days. (3) Incidence = 0.012/year. (4) Case-fatality rate = 0.008. (5) Disability score: DF = 0.81, DHF = 0.85. (6) Treatment cost: DF = US$4.29, DHF = US$139. (7) Vector control costs: not stated. (8) Vaccine: Two-dose regimen; 95% effective; 85% vaccine coverage; 90% public market @ US$0.50/dose + US$0.05/syringe + US$3.50/healthcare contact and other vaccine related costs; 10% private market @ US$10/dose + US$0.10/syringe + US$7.00/healthcare contact and other vaccine related costs.	Dengue vaccination program cost = US$ 81.7 million/year: Vaccination would save US$72.7 million in treatment and 182,000 DALYs¶ /year resulting in a net cost of US$ 9.0 million/year. If vaccination reduced vector control costs by one-third, vaccination would be cost saving.
*VECTOR CONTROL*					
Arthur D. Little Incorporated; I^13^	CBA; 1960–1971; Americas	Vertical vector control; eradication of *Aedes aegypti*	Direct; indirect; vector control; impact on tourism	(1) Outcomes evaluated: reduction in DF and death. (2) Duration: mild = 1 day, severe = 4 days. (3) Incidence = 0·003/year. (4) Case-fatality rate = 0·0002. (5) Treatment cost: severe = US$5. (6) Vector control costs = US$210 million/year. (7) Cost of eradication = US$400 M.	Eradication of *Aedes aegypti* is worthwhile; both dengue and yellow fever may be controlled.
McConnell; IV^33^	CEA; 1984–1994; Puerto Rico	Vertical vector control (larvicide); none	Direct; indirect	(1) Outcomes evaluated: reduction in DF, DHF, and death. (2) Duration: non-hospitalized = 4 days, hospitalized = 14 days. (3) Incidence: Puerto Rico in the years 1983–1989 using multiplication factors: < 18 years = 10 × reported cases; ≥ 18 years = 27 × reported cases. (4) Case-fatality rate = 0.00025. (5) Treatment cost: non-hospitalized = US$96, hospitalized = US$1,389. (6) Statistical value of life = US$3.3 million.	In Puerto Rico, larval control programs that reduce dengue transmission by 50% and cost less than US$2.50 per person will be cost-effective.
Osaka; IV^39^	Cost comparison;‖ 1997; Vietnam	Vertical vector control (ultra low volume pesticide spraying); insecticidal aerosol cans given to residents living in proximity to an identified case	Direct	Outcomes evaluated: reduction in prospectively measured DHF incidence; total cost of the two interventions.	There was a statistically significant lower number of DHF cases in the study area using aerosol cans compared with the area receiving ultra low volume spraying (56 vs. 89 cases) at a lower cost (US$393 vs. US$553).
Baly; II^14^	CEA; 2000–2002; Cuba	Vertical vector control; Vector vertical control + community involvement	Direct	(1) Outcome evaluated: reduction in the number of vector breeding sites. (2) Value of unpaid community worker time was valued at the same rate that a similar type of employment in the government sector.	A program that combined both vertical and participatory methods was more cost effective than vertical control alone: US$1508 vs. US$1767 in the study area.
Suaya; I^45^	CEA; 2001–2005; Cambodia	Vertical vector control (larvicide); none	Direct; indirect	(1) Outcomes evaluated: reduction in DF, DHF, and death. (2) Duration: non-hospitalized = 5.5 days, hospitalized = 9 days. (3) Incidence: Cambodia in the years 2001–2005 using multiplication factor (ambulatory cases = 4 times total reported hospitalized cases. (4) Disability score: DF = 0.19, DHF = 0.15 (however authors used a reversed scale where 0 = death and 1 = perfect health; these scores are equivalent to the 0·81 and 0·85 for DF and DHF, respectively, that most previous authors have used). (5) Treatment cost: DF = US$6.96, DHF = US$57.92. (6) Death averted = 34 DALYs. (7) If no intervention had occurred in intervention area, the incidence of disease in the study area would be the calculated annual average percentage of cases from national surveillance reported in the study area (e.g., this is an ecologic study).	The intervention reduced the number of dengue cases and deaths by 53%. It averted 2,980 hospitalizations, 11,921 dengue ambulatory cases, and 23 dengue deaths, each year, saving of 997 DALYs per year.
Orellano; II^38^	CBA; 2007; Argentina	Vertical vector control; none	Direct; indirect; intangible benefits	(1) Outcomes evaluated: reduction in DF and DHF. (2) Treatment cost: estimated based on local standard of care. (3) Total cases averted were estimated using a calculated baseline expected incidence of 0.005 with an expected DHF/DF ratio of 0.1 based on data published by the Pan American Health Organization for 2006.	In a non-endemic country at risk for dengue outbreaks with an expected incidence of at least 0.029 including DHF cases, a vertical vector control program is cost-beneficial. In the study area, 1,358 cases were averted, savings of US$58,885.**
Tun-Lin; II^48^	Cost comparison;¶ 2007; Kenya, Mexico, Myanmar, Peru, Philippines, Thailand, Venezuela, Vietnam	Targeted vs. non-targeted vector control (larvicide and larval source reduction)	Direct	(1) Outcomes evaluated: Larval indices. (2) This was a non-inferiority study in which a difference of ≤ 1 pupa per person or < 10% from baseline incidence was not considered significant. (3) Range of vector infestation at baseline was not adjusted for by design.	(1) First dengue economic study that included Africa. (2) Multi-country cluster randomized trial. (3) Costs were monitored prospectively across the sites and reported individually.

* See Table 1.

† Study types: CBA = cost benefit analysis; CEA = cost effectiveness analysis.

‡ DHF = dengue hemorrhagic fever.

§ US$ = Unites States dollars.

¶ DALYs = disability adjusted life-years.

‖ The authors reported the difference in cost.

** Converted to US$.

**Table 4:** Dengue health economic expert panel opinion of the priority,* timing,† and perceived importance‡ of additional dengue health economic studies to audiences with interest in a dengue vaccine

	Disease burden		Budget impact of vaccine implementation	Comparative analyses	Stated preference research
	Without monetization	With monetization (cost of illness)			
Need-based priority	1	2	2	1	3
Audience					
Donors§	S+	M0	M+	M+	S+
Vaccine manufacturers	S+	M+	M+	M+	S+
Public health community¶	S+	M0	M–	M+	M0
Private healthcare insurers	L0	S+	M+	M+	M+
Governments & advisory bodies	M+	S0	M+	M+	M+
Clinician organizations	L0	M–	L–	M0	M–
Healthcare providers	L0	M–	L–	M0	M–
Consumers‖	L0	M–	L–	M0	L–

* Need-based priority was rated numerically, 1 = urgent, 2 = needed, 3 = optional.

† Timing or event horizon for commencing new studies over the next 5 years; the results to be most useful: S = Short term (1–2 years), M = Midterm (2–4 years), L = Long term (5–6 years).

‡ Perceived importance to various audiences with interest in a dengue vaccine was rated as follows: (+) = Higher interest, 0 = Medium interest, (–) Lower interest.

§ Donors are those groups providing funding, e.g., development banks.

¶ The public health community includes non-governmental organizations, the World Health Organizations, national and local ministries of health, etc.

‖ Consumers of a dengue vaccine include the general public including special groups (e.g., military, travelers).
